# Quality Evaluation and Chemical Markers Screening of *Salvia miltiorrhiza* Bge. (Danshen) Based on HPLC Fingerprints and HPLC-MS^n^ Coupled with Chemometrics

**DOI:** 10.3390/molecules22030478

**Published:** 2017-03-17

**Authors:** Wenyi Liang, Wenjing Chen, Lingfang Wu, Shi Li, Qi Qi, Yaping Cui, Linjin Liang, Ting Ye, Lanzhen Zhang

**Affiliations:** School of Chinese Materia Medica, Beijing University of Chinese Medicine, Beijing 100102, China; lwy1054289310@163.com (W.L.); sdcwjing@163.com (W.C.); fanglingwu@163.com (L.W.); lishi816@126.com (S.L.); cici_jiayou@163.com (Q.Q.); 20150931830@bucm.edu.cn (Y.C.); 15830243649@163.com (L.L.); m18810820793@163.com (T.Y.)

**Keywords:** *Salvia miltiorrhiza*, fingerprint, chemometric, HPLC-MS^n^, chemical marker, quality evaluation

## Abstract

Danshen, the dried root of *Salvia miltiorrhiza* Bge., is a widely used commercially available herbal drug, and unstable quality of different samples is a current issue. This study focused on a comprehensive and systematic method combining fingerprints and chemical identification with chemometrics for discrimination and quality assessment of Danshen samples. Twenty-five samples were analyzed by HPLC-PAD and HPLC-MS^n^. Forty-nine components were identified and characteristic fragmentation regularities were summarized for further interpretation of bioactive components. Chemometric analysis was employed to differentiate samples and clarify the quality differences of Danshen including hierarchical cluster analysis, principal component analysis, and partial least squares discriminant analysis. Consistent results were that the samples were divided into three categories which reflected the difference in quality of Danshen samples. By analyzing the reasons for sample classification, it was revealed that the processing method had a more obvious impact on sample classification than the geographical origin, it induced the different content of bioactive compounds and finally lead to different qualities. Cryptotanshinone, trijuganone B, and 15,16-dihydrotanshinone I were screened out as markers to distinguish samples by different processing methods. The developed strategy could provide a reference for evaluation and discrimination of other traditional herbal medicines.

## 1. Introduction

The dried root of *Salvia miltiorrhiza* Bge. has been one of the most common traditional herbal medicines for a long time. It is called ‘Danshen’ in China which was first documented in the Chinese oldest pharmaceutical monograph, *Shennong’s Classic of Materia Medica* (Shennong Bencao Jing). As a popular medicine in clinical practice, Danshen plays an important role in the treatment of cardio-cerebrovascular diseases, chronic liver disease, cancer, and osteoporosis [1,2,3,4].

Two groups of the major representative constituents were been isolated from Danshen, which are hydrophilic phenolic acids and lipophilic diterpene quinones, containing danshensu (DSS), protocatechuic aldehyde (PAL), rosmarinic acid (RA), lithospermic acid, salvianolic acid B (SAB), dihydrotanshinone I (DI), cryptotanshinone (CT), tanshinone I (TI), and tanshinone IIA (IIA). Modern pharmacological studies have indicated that these chemicals have various biological activities, such as anti-myocardial ischemia [5], anti-cerebral ischemia [6,7], anti-atherosclerosis [8], anti-tumor [9,10,11], anti-thrombosis [12], anti-hepatocyte injury [13,14], renal protecting [15], anti-inflammatory [16,17,18], etc. The synergistic action of these various components is considered to be responsible for the therapeutic effects of Danshen.

Danshen is used extensively as a raw material in the pharmaceutical and natural product industries. However, decreasing resources and insufficient active ingredients have become more and more serious, which have a negative impact on the internal quality and clinical application of Danshen [19,20]. In this case, a reliable and comprehensive methodology should be developed to validly evaluate their quality.

As is known to all, the quality of traditional herbal medicines is closely related to the concentrations of their active ingredients. Because of multiple and synergistic components, we cannot choose only several specific components as essential criteria. Therefore, it is necessary to establish a multi-criteria quality evaluation system. Chemical fingerprint analysis method was proposed and accepted for the identification of authenticity, differentiation of origin, and evaluation of quality of herbal medicines and related products, which could display the holistic chemical profile obtained by various analytical techniques [21,22,23,24,25,26]. Recently, chemometrics have attracted increasing attention with the development of data mining methodologies, which could simplify complex data and find hidden information. Hierarchical cluster analysis (HCA), principal component analysis (PCA), and partial least squares discriminant analysis (PLS-DA) have been applied to discriminate the class and evaluate the quality of traditional Chinese medicines in existing studies [27,28,29,30]. There are some reports about fingerprint analysis of Danshen to characterize the whole chemical profile [31,32,33,34,35], but in those studies thorough chemometric analysis has not been performed on complex and rich chromatographic peak data to assess their quality, and chemical markers have not been picked as the most significant variables to distinguish Danshen samples of different quality. In addition, a few constituents were inferred and identified by LC-MS in Danshen, and the characteristic fragment information and fragmentation regularities were scattered across several studies. A more complete identification and analysis are required for further interpretation of the bioactive components and Danshen evaluation.

In this present study, 25 Danshen samples were collected. Chemical fingerprints were constructed by a high performance liquid chromatography with photodiode array detection method (HPLC-PDA) with simultaneous separation of phenolic acids and tanshinones. Then a technique, high performance liquid chromatography coupled with electrospray ionization hybrid linear ion trap-Orbitrap mass spectrometry (HPLC-ESI-LQT-Orbitrap/MS), was used for chromatographic peaks identification and mass fragment information characterization. Based on the fingerprint data, HCA, PCA and PLS-DA were undertaken to discriminate samples of different quality. The potential candidate markers were screened out which have the most influence on the quality separation among different groups of samples. This paper provided an objective and effective method of HPLC fingerprinting and component identification coupled with chemometrics for quality evaluation of Danshen.

## 2. Results and Discussion

### 2.1. Optimization of Extraction Conditions

In order to extract the co-existing water-soluble and fat-soluble ingredients from Danshen sufficiently and conveniently, the extraction conditions were optimized through single-factor tests such as solvent, extraction method, and duration. Different concentrations of methanol or ethanol were tested containing 10, 30, 50, 70, and 90% methanol; and 10, 30, 50, 70, and 90% ethanol. By comparing the number of chromatographic peaks and peak areas with the extraction of the different solvents, it was clear that, when 70% methanol was employed, the peaks and peak areas reached the highest values. Therefore, 70% methanol was selected as the extraction solvent. After extraction methods, including reflux and ultrasonication, were investigated. We found that there was no difference for the extraction of water-soluble ingredients between the two methods. However, compared with ultrasonic extraction, the concentrations of tanshinones were higher by reflux extraction. Thus, reflux extraction was regarded as the most suitable method. Finally, the different reflux times (0.5, 1, 1.5, 2 h) were performed to test their impact on the extraction efficiency. When the extraction time is equal to or greater than 1 h, the contents of two kinds of components were no longer increased. The results showed that all contents were almost extracted completely within 1 h. The above experiments suggested that samples were optimally extracted by refluxing with 20 mL of 70% methanol for 1 h.

### 2.2. Methodology Validation

The analytical method was validated for precision, repeatability, and stability. Peak 11 was chosen as a reference substance (SAB) to calculate relative retention times (RRTs) and relative peak areas (RPAs) of common peaks in all samples, because this peak existed in all chromatograms, which was present in the middle of the chromatogram with maximum content. In precision testing, six consecutive injections of one sample solution were analyzed on the same day. Precision of RRTs and RPAs did not exceed 0.525% and 2.17% in RSD, respectively. Six independent samples were extracted and determined in parallel for the evaluation of repeatability. The RSD of RRTs was less than 1.52% and the RSD of RPAs was less than 2.51%. The stability was assessed by measuring the one sample solution stored at 4 °C after 0, 2, 4, 8, 12, and 24 h. The RSDs of RRTs and RPAs of the common peaks were no more than 0.982% and 2.42%, respectively. These results indicated that the method established for the fingerprint analysis of Danshen is stable and reliable.

### 2.3. Chemical Fingerprint Establishment and SA

**Figure 1:**
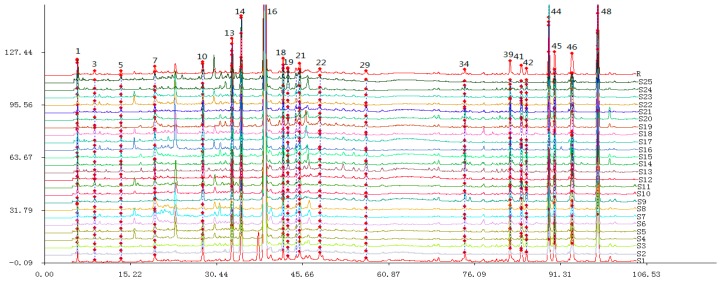
HPLC fingerprints of different Danshen samples. Peak numbers are based on Table 2.

The fingerprints of Danshen samples were established under the HPLC conditions and then analyzed by Similarity Evaluation System*.* The simulative mean fingerprint R was generated, as well as the 21 common components that could be observed in fingerprints (Figure 1). Then, the determined common peaks were tentatively identified using HPLC-MS^n^ technology and standard substances in the next section. 

The sample HPLC fingerprints were compared with the simulative mean fingerprint R to calculate the similarity correlation coefficients, of which all values were in the range of 0.943 to 0.999 (Table 1). This showed that there were no significant differences in the type of chemical composition among various samples. According to the relevant regulations of SFDA [22], all collected samples were qualified due to their correlation coefficients being more than 0.900, and the established simulative mean fingerprint could be used as a standard fingerprint of Danshen samples. The fingerprint chromatography of different samples is different, and we could get the similarity values of each fingerprint by comparing each fingerprint with the standard fingerprint, which helps us to assess the quality of samples in the future. The peak area (PA) of each common peak could reflect the content of the corresponding active constituent and semi-quantitatively express the chemical properties of these samples. The RSDs of PAs of 21 common peaks were calculated between 23.4% and 81.4%. The total PA in each chromatographic fingerprint was also calculated and its RSD was 24.1%. These results indicated that the contents of active ingredients were quite different which might result in the quality difference, although chemotype was very similar between different samples. Then the pattern recognition methods were employed to assess the variation in quality.

### 2.4. Identification of Chemical Components in Danshen

The HPLC-MS^n^ technique was applied for analysis and identification of the 21 common constituents from Danshen samples. In subsequent experiments, the ESI negative ion mode was more suitable for the water-soluble phenolic compounds while positive ion mode was set better for the fat-soluble tanshinones which could achieve high ionization efficiency and sensitive signal response. The deprotonated molecule [M − H] or protonated molecule [M + H]^+^ was selected as the precursor ion for collision-induced dissociation (CID) fragmentation to produce MS/MS product ion spectra. The most prominent product ion was then selected for further MS^n^ analysis.

**Figure 2:**
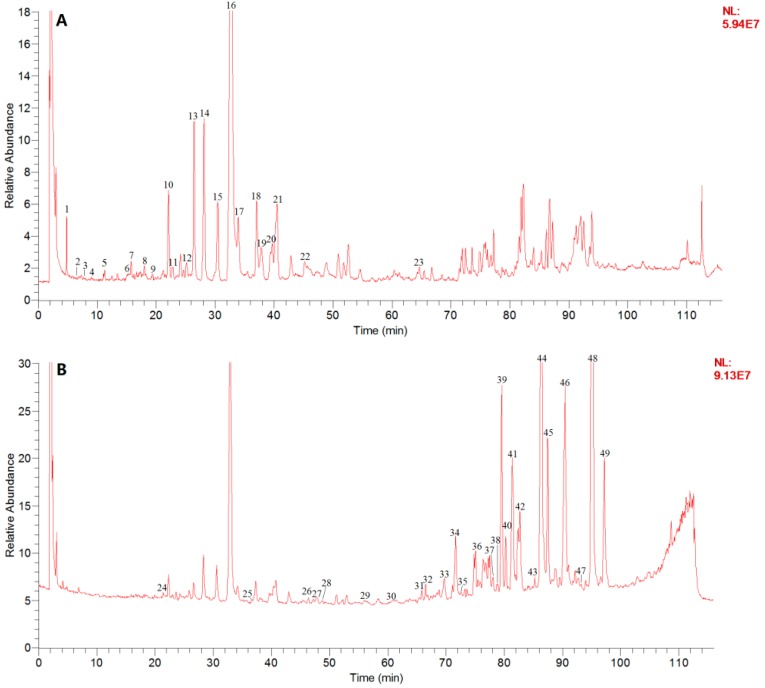
(**A**) TIC chromatogram in negative mode and (**B**) TIC chromatogram in positive mode of Danshen. The peak numbers are based on Table 2.

A total of 49 compounds, including 26 phenolic acids and 23 diterpenoid quinones, were deduced and identified (Table 2). Eight of the identified compounds were authenticated by comparison with reference standards including DSS, PAL, RA, SAB, DI, CT, DI, and IIA. The others were tentatively judged by interpreting their mass spectral behavior and comparing them with related literature data [36,37,38,39,40,41,42,43,44]. The total ion current (TIC) chromatograms are shown in Figure 2.

**Figure 3:**
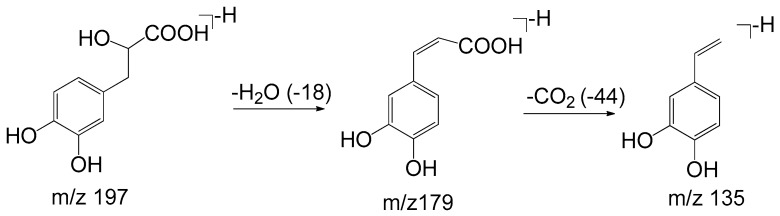
The proposed fragmentation pathway of Danshensu.

**Figure 4:**
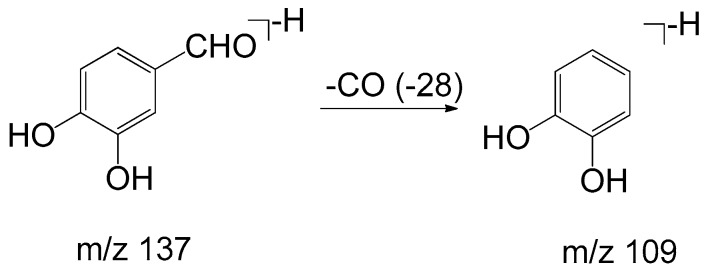
The proposed fragmentation pathway of protocatechuic aldehyde.

**Figure 5:**
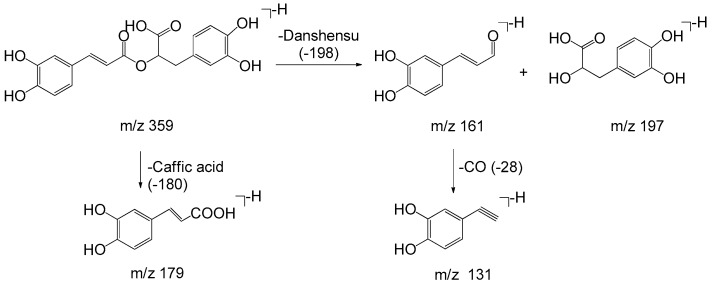
The proposed fragmentation pathway of rosmarinic acid.

**Figure 6:**
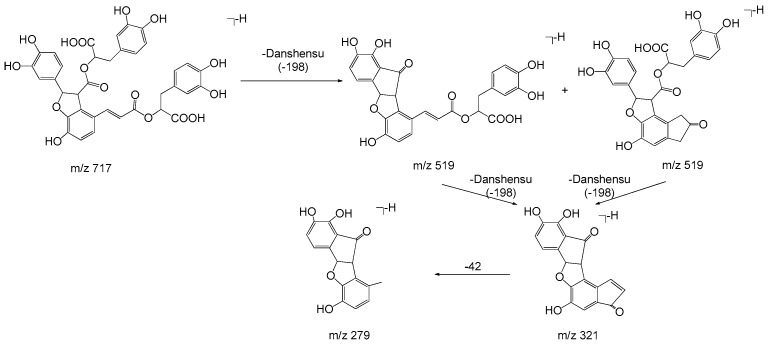
The proposed fragmentation pathway of salvianolic acid B.

**Figure 7:**
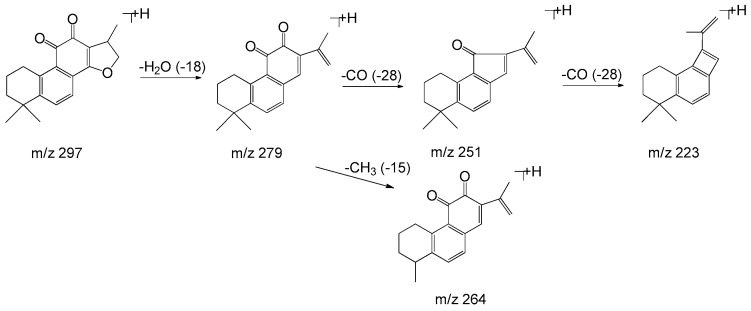
The proposed fragmentation pathway of cryptotanshinone.

**Figure 8:**
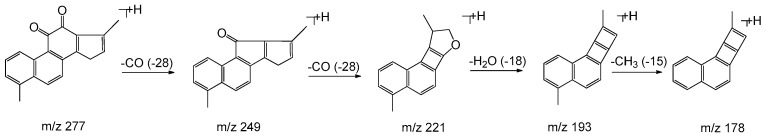
The proposed fragmentation pathway of tanshinone I.

**Figure 9:**
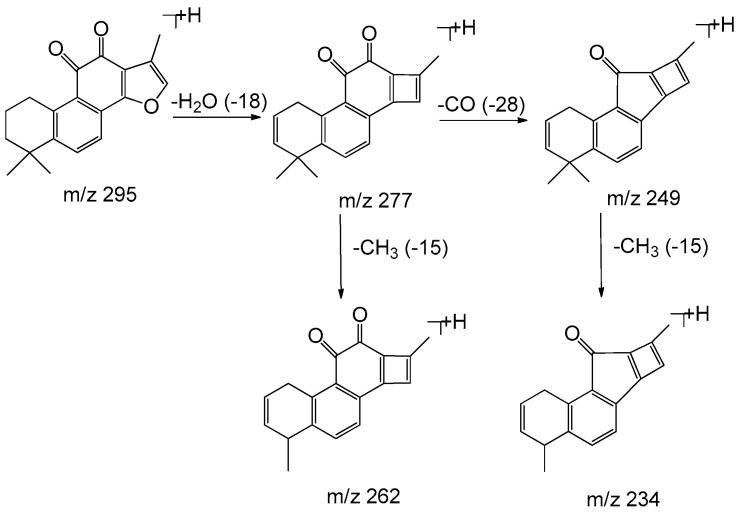
The proposed fragmentation pathway of tanshinone IIA.

Monomers of phenolic acids include DSS, caffeic acid, protocatechuic acid, and PAL which have only phenyl group. The [M − H]⁻ ion at *m*/*z* 197 of DSS produced [M − H − H_2_O]⁻ (*m*/*z* 179) and [M − H − H_2_O − CO_2_]⁻ (*m*/*z* 135) due to the existence of hydroxyl and carboxyl group, and the [M − H]⁻ ion at *m*/*z* 137 of PAL produced the fragment of [M − H − CO]⁻ (*m*/*z* 109). As for dimers, trimers, and tetramers such as RA and SAB, their spectra exhibited [M − H − 198]⁻, [M − H − 198 − 180]⁻, and [M − H − 198 − 198]⁻ ions corresponding to the loss of DSS (198 Da) and caffeic acid (180 Da), respectively. The fragment ions of tanshinones are mainly involved in the loss of a molecule of H_2_O [M + H − 18]^+^ due to enolic rearrangement of a ketonic group. Further loss of CO from these fragment ions gave rise to [M + H − 28]^+^. Besides, the prominent ions of [M + H − 18 − 15]^+^ or [M + H − 28 − 15]^+^ were further formed by loss of a methyl group. The proposed fragmentation pathways of the typical phenolic acids and tanshinones are illustrated in Figure 3, Figure 4, Figure 5, Figure 6, Figure 7, Figure 8 and Figure 9.

### 2.5. Chemometric Analysis

#### 2.5.1. HCA

**Figure 10:**
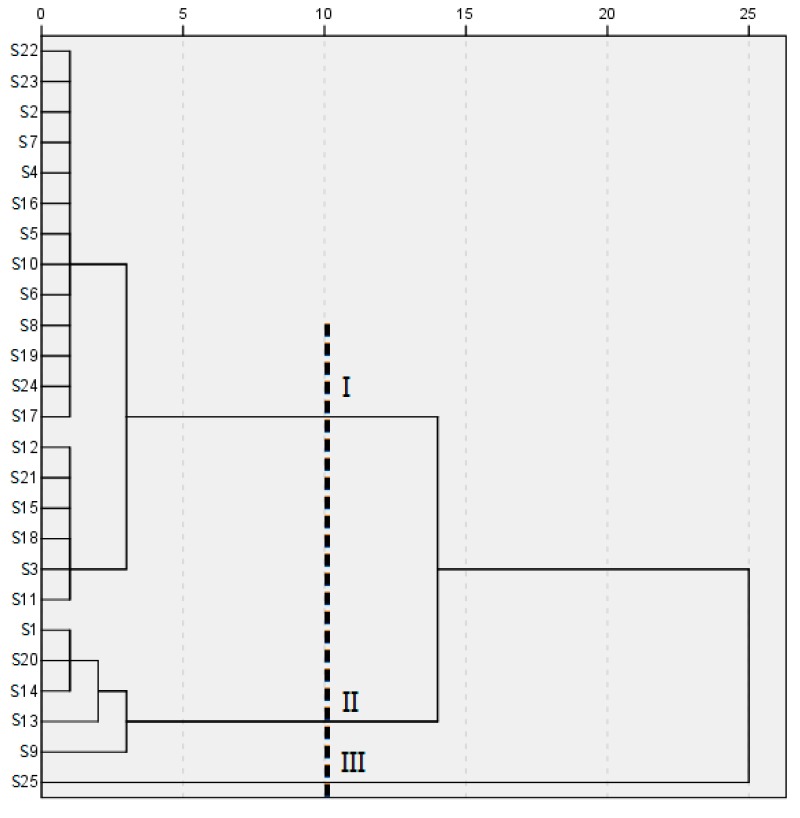
HCA dendrogram of different Danshen samples.

In order to evaluate resemblance and dissimilarities of Danshen samples based on the fingerprint data, HCA was performed which could divide tested samples into different categories. Individuals within the same category should have the highest possible homogeneity and the highest possible heterogeneity should exist between different categories. Figure 10 displays the dendrogram which was generated with PAs of the 21 common components from 25 fingerprints and formed a 21 × 25 matrix. These samples were sorted into three clusters, reflecting the difference in quality of Danshen samples from the viewpoint of active ingredient contents. With the purpose of investigating the reasons for sample classification, we had studied the grouped samples one by one in every detail from its marks (the collection date, cultivation methods, geographic origins, and processing methods) to total PA of common peaks in each fingerprint which could reveal the total content of active constituents. From the dendrogram, samples were mixed together and not grouped strictly on the basis of their origins. Yet samples processed by sweating or sun curing were grouped into the same class (cluster I) and most of the samples dried in the shade were in cluster II except S15 and S19. The sample dried in the oven belonged to cluster III. It was suspected that sample classification might be relevant to processing method rather than the origin. Then all samples were treated by different processing methods respectively, including sun curing, shade drying, oven drying, and sweating, to explore the impact on metabolites caused by geographical origins and processing methods. The results showed that the samples processed by the same method were similar on fingerprints and total PA of common peaks and we could rule out the influence of origin on sample quality. However, there were significant differences between samples which were from the same location but treated by different processing methods, it indicated the processing method could affect the internal chemical profile. In addition, the total content of common ingredients was the highest in cluster II, followed in decreasing orders by cluster III and cluster I. Therefore, we could come to the conclusion that the processing method had a more obvious impact on sample classification than the geographical origin, it induced the different content of bioactive compounds and finally lead to different qualities. Next, the other chemometric analysis was taken into consideration to measure the degree of the similarity among the samples in the same cluster, and pick a few key chemical markers for explaining sample variation.

#### 2.5.2. PCA

**Figure 11:**
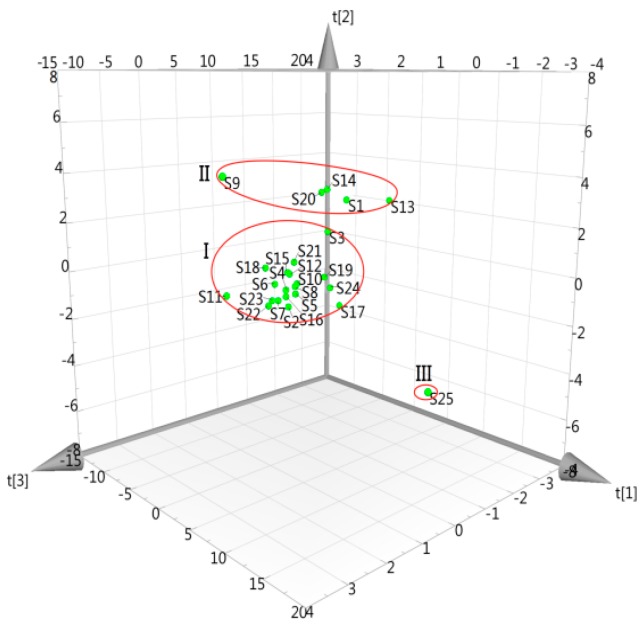
3D score plot of PCA on the first three PCs for Danshen samples.

The fingerprints are usually wide data matrices, characterized by a very large number of variables. PCA is a simple non-parametric method widely used for sample categorization further by data compression and information extraction [45]. It could fit a small number of underlying factors called principal components (PCs) which were unique, mutually exclusive, and accounted for as much of the variable information as possible [28,46]. In accordance with Eigenvalue >1, the first three PCs were extracted and explained 71.0, 18.0, and 5.29% of the total variability, respectively. All three PCs could accumulate about 94.3% data variance, and the roles of other PCs were insignificant. The 3D score plot of the first three PCs is showed in Figure 11, where each axis represents a PC and all samples could be separated into three groups. S25 was outside the scope of Hotelling’s T2 ellipse, which meant notable differences with others due to the different processing methods. The dissimilarities of Danshen samples were observed intuitively, indicating that the contents of common ingredients were different in spite of similar chemotypes. Moreover, the samples in cluster I were scattered in a larger region compared with those of cluster II and III. It demonstrated that the products of cluster I had a larger quality fluctuation than those by other processing methods. In our study, the PCA classification results were consistent with HCA results and this method provided a more reliable evidence for the discrimination of Danshen. However, it was still not clear which major compounds caused the classification of these samples. Therefore, a PLS-DA technique development is necessary to find out definite indexes of describing the differences.

#### 2.5.3. PLS-DA

PLS-DA was conducted as a supervised recognition pattern technique to optimize classification and screen out the main chemical markers responsible for the sample variation. At the time of establishing the model, it is very important to select the optimal number of latent variables, which could avoid over-fitting and improve the reliability and accuracy of the modeling results. By analyzing the relationship between *X* variables and *Y* variables, samples can be also sorted into diverse groups vividly to confirm the classification results by means of the HCA. The profile of variables important for the projection (VIP) suggests the contributions of each *X* variable and helps to find out the main chemical markers for categories differentiation [47].

**Figure 12:**
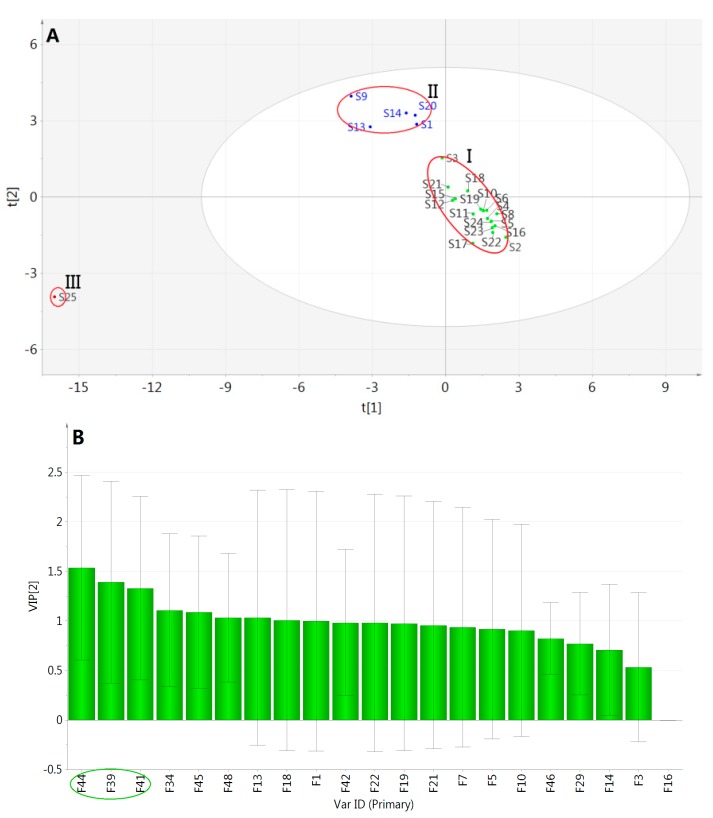
(**A**) 2D score plot of the two latent variables and (**B**) VIP plot for Danshen samples based on PLS-DA.

The selected optimal number of latent variables was two for building a PLS-DA model. The values of R2X, R2Y, and Q2 were 0.890, 0.886, and 0.746, respectively, which showed that this model had better fitting and predictive ability in data processing. Then, a permutation test was performed to validate the model and the result of 200 permutations was that the vertical intercept values of R2 and Q2 were −0.00537 and −0.261 respectively, indicating that the established model avoided an over-fitting problem and improved the forecasting accuracy effectively. Based on a 2D score plot of the two latent variables (Figure 12A), the test samples were excellently divided into three categories which were in agreement with HCA and PCA results. The VIP profile (Figure 12B) was constructed to measure the importance of *X* variables on sample discrimination, and the VIP values of many variables were greater than 1, which figured that these *X* variables were rather meaningful for distinguishing between Danshen samples. Among them, peaks 44, 41, and 39 might have more substantial influence which represented cryptotanshinone, trijuganone B, and 15,16-dihydrotanshinone I, respectively. Then the total PA of the above three components in each fingerprint were analyzed, which could reflect the total content of marker constituents. It was found that the values in cluster II were the highest, followed in decreasing orders by cluster III and cluster I, indicating that there were more obvious differences in the contents of these components among different clusters than others’ contents. Thus, the three components could be considered as the chemical markers to discriminate Danshen samples by different processing methods.

## 3. Materials and Methods

### 3.1. Materials and Reagents

DSS, PAL, RA, SAB, DI, CT, TI, and IIA were purchased from Chengdu Must Biotechnology Co., Ltd. (Chengdu, China). Their purity was above 98% by HPLC analysis.

HPLC grade acetonitrile and formic acid (Fisher, Fair Lawn, NJ, USA) were used for HPLC analysis. Distilled water was supplied by Watsons Group Co., Ltd. (Beijing, China). Methanol and other reagents were all of analytical grade and obtained from Beijing Chemical Factory (Beijing, China).

The fresh roots of annual Danshen were collected in later October from different provinces in China and processed by the local and common drying methods (Table 1). S1, S9, S13–15, S19–20 were placed in the shade to dry. S16–18 from Sichuan Province were treated by sweating method, the procedure is as follows: dry the fresh roots in the sun to a semi-dry state, then pile up them for four days to make the internal moisture spillover, when the inner core of the roots becomes purplish red, intermediate products are spread out to dry totally in the sun. S25 from Anhui Province was heated in an oven at 60 °C. Others were dried by the sunlight directly. All dry Danshen samples were authenticated by Prof. Lanzhen Zhang, School of Chinese Materia Medica, Beijing University of Chinese Medicine. Then these materials were cut into small pieces and further crushed into powder, passed through a 40-mesh (0.420 mm) sieve, and stored in a brown desiccator at laboratory temperature (25 °C approx.) before analysis.

### 3.2. Instrumentation and Chromatographic Conditions

#### 3.2.1. HPLC Instrumentation and Chromatographic Conditions

A Waters e2698 HPLC system (Waters, Milford, MA, USA) was equipped with a photodiode array detector, a quaternary solvent delivery pump, an online degasser, a column temperature controller, and an auto sampler. System control and data analysis were processed with Empower 3 software (Waters). The separation was performed on a DIKMA Diamonsil C18 column (250 mm × 4.6 mm, 5 µm).

The mobile phase was composed with solvent A (acetonitrile) and solvent B (0.2% aqueous formic acid) with a gradient elution program: 0–15 min, 10–18% A; 15–35 min, 18–25% A; 35–45 min, 25–30% A; 45–55 min, 30% A; 55–75 min, 30–55% A; 75–85 min, 55–67% A; 85–100 min, 67–79% A; 100–101 min, 79–90% A; 101–110 min, 90% A. The constant flow rate was 1 mL/min and the column was maintained at 30 °C. The injection volume was 20 µL and the detection wavelength was set at 270 nm.

#### 3.2.2. HPLC-MS^n^ Instrumentation and Chromatographic Conditions

The mass analysis was performed on an LTQ-Orbitrap mass spectrometer which was equipped with a Thermo Accela 600 HPLC system and an ESI source (Thermo Fisher Scientific, Bremen, Germany). The chromatographic condition was same as the described in Section 3.2.1. The source parameters in negative and positive ionization mode were as follows: source voltage, −3.0 kV(−)/4.0 kV (+); capillary voltage, −35 V(−)/25 V(+); tube lens voltage, −100 V(−)/100 V(+); capillary temperature, 350 °C; vaporizer temperature, 300 °C; sheath gas, 30 arbitrary units; auxiliary gas, 10 arbitrary units. The sample solution was first analyzed in full MS mode with a resolution of 30,000. The successive analyses were done in data-dependent MS^n^ mode, in which the three most intense ions were isolated and fragmented by CID with normalized collision energy of 35% and an isolation width of 2 *m*/*z* units. The mass scan range was from *m*/*z* 100 to 1000. For avoiding repeated data collection, the dynamic exclusion was activated with exclusion duration of 60 s, and the exclusion was repeated for 30 s with the repeat count at 5. Data were processed by Xcalibur 2.1 software (Thermo Fisher Scientific). Before the analysis, the external calibration was carried out and the measured masses were within 5 ppm of the theoretical masses.

### 3.3. Preparation of Sample and Standard Solutions

Each powder sample (0.2 g) was weighed and extracted with 20 mL of 70% methanol in a refluxing bath for 1 h. After centrifugation at 13,000 rpm for 10 min, the supernatant was transferred into a HPLC sample vial and injection. The mixed standard solution was prepared at concentrations of 81.0 µg/mL DSS, 9.74 µg/mL PAL, 28.2 µg/mL RA, 33.2 µg/mL SAB, 7.50 µg/mL DI, 6.60 µg/mL CT, 10.8 µg/mL TI, and 5.71 µg/mL IIA in 70% methanol. These solutions were stored at 4 °C.

### 3.4. Data Analysis

#### 3.4.1. Similarity Analysis (SA)

All chromatographic data of 25 different batches of samples was mined and analyzed. Similarity analysis was performed by professional software—*Similarity Evaluation System for Chromatographic Fingerprint of Traditional Chinese Medicine* (Version 2004A, Chinese Pharmacopoeia Committee). The simulative mean fingerprint R of Danshen was generated and the similarity values of these samples with mean fingerprint R were calculated. Then the common peaks existing in Danshen were observed and the RRT and RPA of each common peak were also calculated.

#### 3.4.2. Chemometric Analysis

In order to find out the quality similarities and differences between various samples, chemometric analysis was performed including HCA, PCA, and PLS-DA. In HCA program, a dendrogram was drawn to characterize the classification results of the samples by Ward’s linkage as the cluster method and squared Euclidean distance as the metric using SPSS 20.0 software (SPSS, Chicago, IL, USA). PCA was carried out by SIMCA-P 13.0 software (Umetrics AB, Umea, Sweden) and SPSS 20.0. The important PCs were exacted on the condition that corresponding eigenvalues were greater than 1. The sample variation could be assessed from the score plot. PLS-DA was processed by the SIMCA-P 13.0 software which helps to screen out the main markers responsible for discrimination.

## 4. Conclusions

In the present research, a comprehensive method was developed combining the HPLC fingerprints and chemical identification with chemometric analysis to discriminate and assess Danshen samples. The chemical fingerprints were established and 21 common peaks were observed among the 25 samples, and these peaks were identified by HPLC-MS^n^ technique. Their characteristic fragment information and fragmentation regularities were summarized in detail for further interpretation of bioactive components. Although there was similar chemical composition between different samples, their contents were very different which might result in the quality difference. Chemometrics were applied to evaluate quality of Danshen—including HCA, PCA, and PLS-DA—and the test samples could be divided into three categories. By analyzing the reasons for sample classification, it was revealed that the processing method had a more obvious impact on sample classification than the geographical origin, it induced the different content of bioactive compounds and finally lead to different qualities. Then three characteristic markers (cryptotanshinone, trijuganone B, and 15,16-dihydrotanshinone I) were screened out as the most significant variables to distinguish Danshen samples by different processing methods. The established approach is reliable, sensitive, and promising for accurate discrimination and quality assessment of Danshen.

## Figures and Tables

**Table 1:** Information summary and similarity values of tested samples.

No.	Origins	Similarities	No.	Origins	Similarites
S1	Shandong	0.972	S14	Shanxi	0.982
S2	Shandong	0.997	S15	Shanxi	0.994
S3	Shandong	0.993	S16	Sichuan	0.997
S4	Shandong	0.999	S17	Sichuan	0.991
S5	Shaanxi	0.998	S18	Sichuan	0.999
S6	Shaanxi	0.999	S19	Jiangsu	0.996
S7	Shaanxi	0.997	S20	Jiangsu	0.973
S8	Henan	0.999	S21	Gansu	0.997
S9	Henan	0.943	S22	Hunan	0.998
S10	Hebei	0.999	S23	Hunan	0.999
S11	Hebei	0.999	S24	Yunnan	0.998
S12	Hebei	0.998	S25	Anhui	0.994
S13	Shanxi	0.975			

**Table 2:** Identification of compounds in Danshen by HPLC-MS^n^.

ID	*t*_R_ (min)	Mass Ion (*m*/*z*)	Data-Dependent MS^n^ Data (*m*/*z*)	Identification
1 ^a^	4.79	[M − H]⁻ 197.0452	MS^2^：178.9395 MS^3^: 134.9681, 106.9022	Danshensu
2	6.19	[M − H]⁻ 153.0191	MS^2^: 109.0295	Protocatechuic acid
3	7.98	[M − H]⁻ 341.1076	MS^2^：178.9334, 160.8835, 112.8926	1-*O*-caffeoyl glucose
4 ^a^	8.96	[M − H]⁻ 137.0242	MS^2^: 109.0310	Protocatechuic aldehyde
5	13.43	[M − H]⁻ 179.0344	MS^2^: 135.0451	Caffeic acid
6	15.10	[M − H]⁻ 683.1226	MS^2^: 665.2238, 621.3198, 441.1573	3,4-Dihydroxy-(1α,3α,4α,5β)-1-carboxy-4-hydroxy-1,3,5-cyclohexanetriyl ester-benzenepropanoic acid
7	15.74	[M − H]⁻ 313.0707	MS^2^: 269.0386, 158.9700, 108.8062	Salvianolic acid F
8	17.97	[M − H]⁻ 539.1178	MS^2^: 521.1625, 495.3337, 359.1708, 341.1323, 297.1190	Yunnaneic acid D/isomer
9	19.34	[M − H]⁻ 521.1287	MS^2^: 498.5744, 477.9366, 359.1708, 341.1323, 297.1190	Salviaflaside
10	22.09	[M − H]⁻ 339.0501	MS^2^: 320.9998, 294.9889, 184.9634	Salvianolic acid G
11	22.76	[M − H]⁻ 735.1541	MS^2^: 716.9385, 537.1756, 519.1987, 555.0787	Hydrosalvianolic acid B
12	25.17	[M − H]⁻ 717.1436	MS^2^: 693.3942, 519.1561, 321.0740, 537.1210	Iso salvianolic acid E
13 ^a^	26.43	[M − H]⁻ 359.0763	MS^2^: 341.1426, 160.8524, 197.0617, 178.8373 MS^3^: 160.9093, 132.8333	Rosmarinic acid
14	28.12	[M − H]⁻ 537.1038	MS^2^: 493.1133, 313.0726, 295.0606	Lithospermic acid
15	28.51	[M − H]⁻ 749.1708	MS^2^: 717.2390, 551.2418, 321.1771	8-Hydroxy-9''-methyl-salvianolate B
16 ^a^	32.67	[M − H]⁻ 717.1426	MS^2^: 519.1459, 321.1666 MS^3^: 359.1635, 322.9468	Salvianolic acid B
17	33.94	[M − H]⁻ 717.1445	MS^2^: 519.1002, 321.1299	Iso salvianolic acid B
18	37.06	[M − H]⁻ 717.1447	MS^2^: 673.0787, 519.1280, 321.0964	Salvianolic acid L
19	38.03	[M − H]⁻ 493.1127	MS^2^: 475.0607, 313.1490, 295.0408	Salvianolic acid A
20	39.86	[M − H]⁻ 731.1599	MS^2^: 713.0930, 533.1845, 335.1095	Methyl salvianolic acid B
21	40.50	[M − H]⁻ 551.1185	MS^2^: 519.1639, 353.0330, 321.0014	Methyl salvianolic acid I/H
22	45.70	[M − H]⁻ 717.6401	MS^2^: 673.2368, 617.6599, 519.1243, 321.1423	Salvianolic acid E
23	64.63	[M − H]⁻ 505.1127	MS^2^: 473.1639, 321.1718	Methyl salvianolate C
24	21.35	[M + H]^+^ 315.1228	MS^2^: 297.0677, 266.9693, 249.0071	17-Hydroxytanshindiol B
25	37.09	[M + H]^+^ 313.1072	MS^2^: 294.9926, 264.9864, 247.0231	Tanshinone A
26	46.34	[M + H]^+^ 297.1122	MS^2^: 279.0668, 250.9774, 223.1107	Tanshinone VI
27	47.88	[M + H]^+^ 297.1127	MS^2^: 279.0222, 269.0689, 251.0355	Danshenxinkun A
28	48.81	[M + H]^+^ 299.1283	MS^2^: 281.0504, 263.0121	15,16-Dihydrotanshinol B
29	56.29	[M + H]^+^ 313.1437	MS^2^: 295.0622, 277.0113, 267.0143	Tanshindiol B
30	60.48	[M + H]^+^ 313.1440	MS^2^: 295.0879, 277.0540, 267.0566, 249.0650	Tanshindiol C
31	65.82	[M + H]^+^ 295.0967	MS^2^: 276.9836, 267.0344, 252.9953,249.0193	3*α*-Hydroxymethylenetanshinquinone
32	66.45	[M + H]^+^ 297.1124	MS^2^: 279.0198, 261.0336	Tanshinol B
33	70.11	[M + H]^+^ 295.0966	MS^2^: 277.0328, 267.0656	Trijuganone A
34	71.64	[M + H]^+^ 311.1276	MS^2^: 293.0745, 267.0956MS^3^: 275.0365, 251.0166	Tanshinone IIB
35	71.86	[M + H]^+^ 311.1279	MS^2^: 293.0213, 275.0118, 267.0738	3*α*-Hydroxytanshinone IIA
36	74.83	[M + H]^+^ 341.1381	MS^2^: 281.0114, 263.0162	Methyldihydronortanshinonate
37	78.02	[M + H]^+^ 309.1119	MS^2^: 281.0635	Tanshinaldehyde
38	78.92	[M + H]^+^ 311.1280	MS^2^: 293.0916, 283.0533, 267.0832	17-Hydroxycryptotanshinone
39 ^a^	79.33	[M + H]^+^ 279.1015	MS^2^: 260.9656, 233.0795	15,16-Dihydrotanshinone I
40	80.23	[M + H]^+^ 297.1483	MS^2^: 269.1145, 279.0749, 251.1033 MS^3^: 251.0895, 241.0596	1-Oxomiltirone
41	81.76	[M + H]^+^ 281.1177	MS^2^: 263.0103, 252.9724, 235.0020 MS^3^: 234.9965, 220.9875	Trijuganone B
42	82.68	[M + H]^+^ 339.1228	MS^2^: 311.0540, 279.0392, 261.1021 MS^3^: 260.9993	Methyl tanshinonate
43	85.19	[M + H]^+^ 267.1381	MS^2^: 249.0448, 224.9753	4-Methylenemiltirone
44 ^a^	86.29	[M + H]^+^ 297.1481	MS^2^: 279.1028, 251.0883, 237.0703 MS^3^: 264.0307, 251.0550, 237.0704,	Cryptotanshinone
45 ^a^	87.42	[M + H]^+^ 277.0855	MS^2^: 249.0238, 231.058.9814	Tanshinone I
46	90.41	[M + H]^+^ 279.1016	MS^2^: 260.9862, 233.0613, 221.0357, 258.9814	1,2-Dihydrotanshinone I /3,4-dihydrotanshinone I
47	93.18	[M + H]^+^ 281.1539	MS^2^: 220.9732, 253.0703, 263.1043, 239.0273	1,2-Didehydromiltirone
48 ^a^	95.04	[M + H]^+^ 295.1327	MS^2^: 277.0313, 249.1205, 235.0146	Tanshinone IIA
49	97.18	[M + H]^+^ 283.1689	MS^2^: 265.0710, 241.0755, 237.0394, 223.0768 MS^3^: 250.0806,237.0400, 223.0643, 195.0449	Miltirone

^a^ Positively identified via comparison with reference standards.
